# Control of Cell Differentiation by Mitochondria, Typically Evidenced in *Dictyostelium* Development

**DOI:** 10.3390/biom3040943

**Published:** 2013-11-11

**Authors:** Yasuo Maeda, Junji Chida

**Affiliations:** 1 Department of Developmental Biology and Neurosciences, Graduate School of Life Sciences, Tohoku University, Aoba, Sendai 980-8578, Japan; 2 Division of Molecular Neurobiology, Institute for Enzyme Research, The University of Tokushima, Kuramoto-cho, Tokushima 770-8503, Japan; E-Mail: jchida@tokushima-u.ac.jp

**Keywords:** differentiation, mitochondria, mitochondrial ribosomal protein S4 (mt-RPS4), tumor necrosis receptor-associated protein 1 (TRAP-1), CN-resistant respiration, prespore-specific vacuole (PSV), *Dictyostelium*, ESC

## Abstract

In eukaryotic cells, mitochondria are self-reproducing organelles with their own DNA and they play a central role in adenosine triphosphate (ATP) synthesis by respiration. Increasing evidence indicates that mitochondria also have critical and multiple functions in the initiation of cell differentiation, cell-type determination, cell movement, and pattern formation. This has been most strikingly realized in development of the cellular slime mold *Dictyostelium*. For example, the expression of the mitochondrial ribosomal protein S4 (*mt-rps4*) gene is required for the initial differentiation. The *Dictyostelium* homologue (Dd-TRAP1) of TRAP-1 (tumor necrosis receptor-associated protein 1), a mitochondrial molecular chaperone belonging to the Hsp90 family, allows the prompt transition of cells from growth to differentiation through a novel prestarvation factor (PSF-3) in growth medium. Moreover, a cell-type-specific organelle named a prespore-specific vacuole (PSV) is constructed by mitochondrial transformation with the help of the Golgi complex. Mitochondria are also closely involved in a variety of cellular activities including CN-resistant respiration and apoptosis. These mitochondrial functions are reviewed in this article, with special emphasis on the regulation of *Dictyostelium* development.

## 1. Introduction

In eukaryotic cells, mitochondria are self-reproducing organelles with their own DNA and play a central role in ATP synthesis by respiration. The normal mitochondrial function is a critical factor in maintaining cellular homeostasis in various organs of the body. Mitochondrial disease and mitochondria-dependent sterility as well as a close relationship between mitochondria and programmed cell death (apoptosis) have been widely recognized as notable events. Understanding the mitochondrial function has been an exciting challenge for many researchers, including cell biologists, biochemists, and physiologists, since its discovery more than 125 years. Unexpectedly, however, the control of cell differentiation by mitochondria and its possible mechanisms has been scarcely focused on. An origin of mitochondrion is believed to be aerobic bacteria that once established a symbiosis with a host cell such as archeabacteria and has been handing over parts of its own genome to the nuclear DNA of the host cell during evolution, thus resulting in failure of existent mitochondria to self-reproduce without help of the nuclear genome.

**Figure 1:**
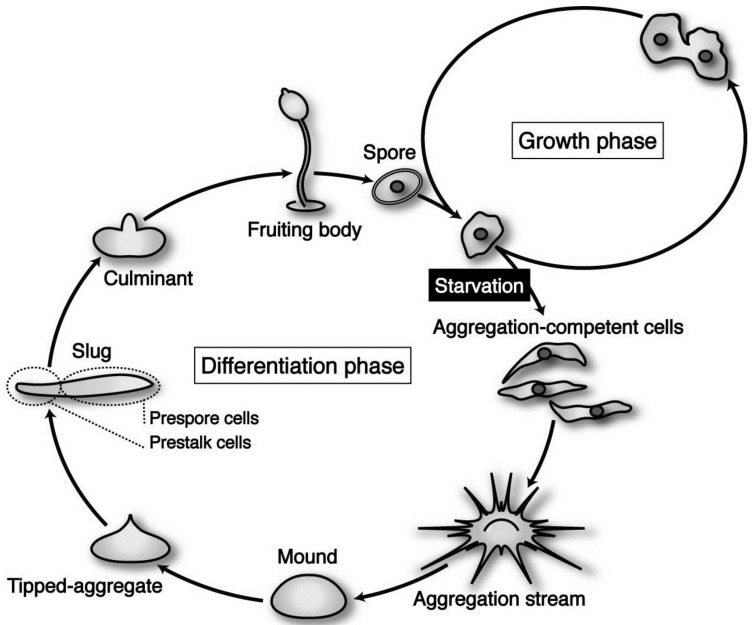
The life cycle of *Dictyostelium discoideum* axenic strain Ax-2. The vegetative cells are usually grown in liquid medium, by means of pinocytotic incorporation of external nutrients. Under natural conditions, its parental strain *D. discoideum* NC-4 grows and multiplies by mitosis at the vegetative phase, phagocytosing nearby bacteria such as *Escherichia coli* and *Klebsiella aerogenes*. Upon exhaustion of nutrients, however, starving cells initiate differentiation, form multicellular structures (aggregates; mounds), and undergo a series of well-organized morphogenesis to construct fruiting bodies, each of which is consisting of a mass of spores (sorus) and a supporting cellular stalk. (see the text for details).

*Dictyostelium discoideum* is a social amoeba whose life cycle consists of two distinct phases—growth and differentiation—that are easily controlled by nutritional conditions. *D. discoideum* (strain Ax-2) cells grow and multiply by mitosis as long as nutrients are supplied (Figure 1). Upon exhaustion of nutrients, however, starving cells initiate differentiation to acquire aggregation competence and form multicellular structures by means of chemotaxis toward 3’,5’-cyclic adenosine monophosphate (cAMP) and ethylenediaminetetraacetic acid (EDTA)-resistant cohesiveness. Subsequently, the cell aggregate (mound) undergoes a series of well-organized movements and zonal differentiation to form a migrating slug. The slug eventually culminates to form a fruiting body consisting of a mass of spores (sorus) and a supporting cellular stalk. At the slug stage, a clear pattern along the anterior–posterior axis is established; prestalk cells, which finally differentiate into stalk cells during culmination, are located in the anterior one-fourth, while prespore cells destined to differentiate eventually into spore cells occupy the posterior three-fourths of the slug (Figure 1). The life cycle of *Dictyostelium* cells is and relatively simple, but it contains almost all of the cellular processes (movement, adhesiveness, differentiation, pattern formation, *etc*.) essential for the establishment of multicellular organization. In basically haploid *Dictyostelium* cells, gene disruptions by homologous recombination are available for analysis of precise gene functions. Insertional mutagenesis by the restriction enzyme–mediated integration (REMI) method has also been established to isolate and characterize intriguing functional genes [1]. Thus *Dictyostelium* is a useful model system for investigating a various aspects of cellular development.

**Figure 2:**
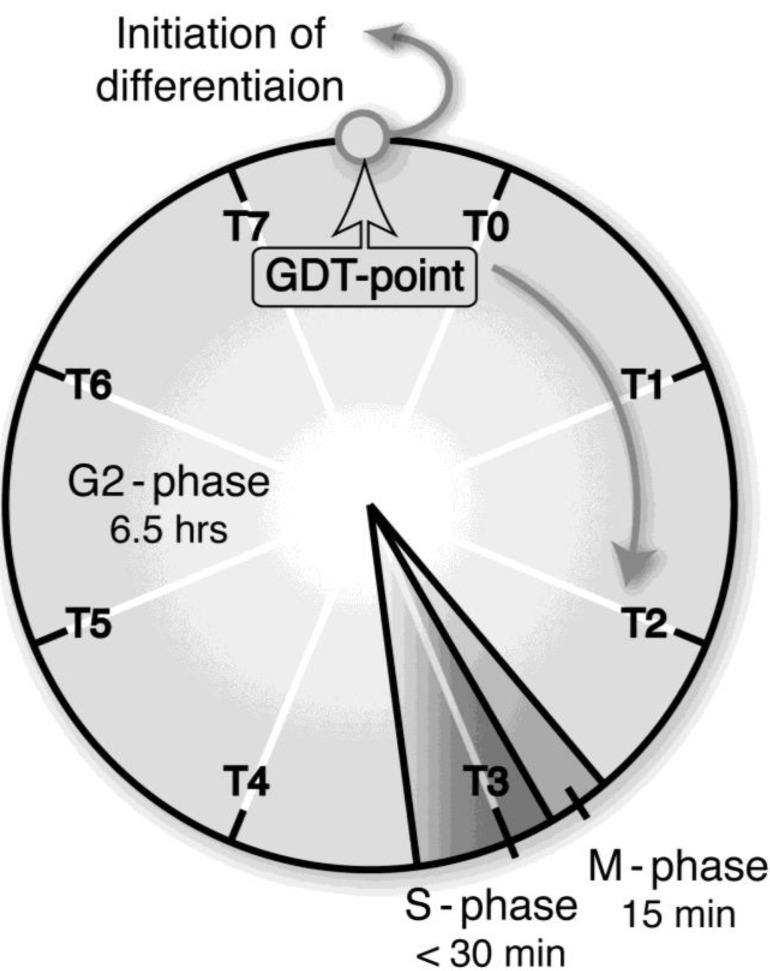
A growth/differentiation checkpoint (GDT point) in the cell cycle of a *Dictyostelium discoideum* Ax-2 cell. The doubling time of axenically growing Ax-2 cells is about 7.2 h and most of their cell cycle is composed of G2-phase with little or no G1-phase and a short period of M- and S-phases. A specific checkpoint (referred to as the GDT point) of GDT is located at the mid–late G2-phase (just after T7 and just before T0). Ax-2 cells progress through their cell cycle to the GDT point, irrespective of the presence or absence of nutrients, and enter the differentiation phase from this point under starvation conditions [2]. T0, T1, and T7 indicates 0, 1, and 7 h, respectively, after a temperature shift from 11.5 °C to 22.0 °C for cell synchrony. The absence of G1 phase in the *Dictyostelium* cell cycle is not so strange, because there is little or no G1 phase in rapidly dividing cells such as animal cells at the cleavage stage, and also in the true slime mold *Physalum* and *Hydra*. (Basically from Maeda [3]).

Growth and differentiation are fundamental characteristics of the cell. In general, they are mutually exclusive but are cooperatively regulated throughout development. Thus, the process of a cell’s switching from growth to differentiation is of great importance not only for the development of organisms but also for malignant transformation, in which this process is reversed. When most cells are terminally differentiated, they must exit the cell cycle. We have precisely specified a critical checkpoint (growth/differentiation transition or GDT point), from which cells start differentiation in response to starvation, in the cell cycle of *Dictyostelium* cells (Figure 2) [2,3]. Accordingly, integration of GDT point–specific events with starvation-induced events is needed to understand the mechanism regulating GDTs. Beyond our imagination, increasing evidence indicates that mitochondria have novel, essential, and multiple functions as the regulatory machinery of the initiation of differentiation, cell-type determination, cell movement and pattern formation, Since these mitochondria-related events have been most strikingly illustrated in the developmental course of *Dictyostelium* cells, they are primarily reviewed in this article.

## 2. Induction of Cell Differentiation by Mitochondrial Ribosomal Protein S4 (mt-RPS4) in Dictyostelium Development

Cell proliferation is finely regulated by extra cellular signals such as growth factors, and there are some checkpoints monitoring the exact progress of the cell cycle, e.g., the G2-phase checkpoint for DNA damage [4] and the M-phase for spindle formation [5,6]. It has been shown that a specific checkpoint regulating the transition from growth to differentiation exists in the G1-phase [7]. Based on much experimental data obtained by synchronized D. discoideum axenic strain (Ax-2) cells, we have succeeded in isolating at least four genes (*quit1* and *dia1*, *2*, *3*) were isolated as being expressed specifically in Ax-2 cells starved just before the GDT point by means of differential plaque hybridization and differential display [8,9,10,11,12].

The coding region of quit1 is identical to that of the cAMP receptor 1 (*carA*) gene [8], a G-protein-linked surface cAMP receptor, exerts a central role in *Dictyostelium* development including cell aggregation; its disruption by homologous recombination and antisense RNA results in the failure of transformed Ax-3 cells to differentiate [13,14], thus providing evidence of the role of CAR1 in the exit of cells into differentiation and also the real existence of the GDT point in the *Dictyostelium* cell cycle. The forced expression of a novel gene, *dia1*, was found to rather impair the progression of differentiation, possibly coupled with the reduced expression of early genes such as the cAMP receptor 1 (*carA*), and the inhibitory effect of forced *dia1* expression is almost completely nullified by externally applied cAMP pulses (Hirose *et al*. 2000). In contrast, antisense RNA-mediated inactivation of *dia1* enhances the initial step of differentiation, as exemplified by precocious expression of *carA* and other early genes [11]. Provided that the *dia1* expression transiently suppresses the progression of differentiation, it is possible that the time difference between cells located at different cell-cycle phases at the time-point of starvation may be shortened, thus allowing both of the T0 (just after the GDT-pint) and T7 (just before the GDT-point) cells to coordinately participate in forming a common aggregate (see Figure 1). The *dia2* gene encodes a novel lysine- and leucine-rich protein (DIA2) with a predicted mass of 16.9 kDa, and the mRNA accumulates in differentiating cells starved just before the GDT point, while there is no detectable expression in vegetatively growing cells [9]. The *dia2* expression pattern during the whole course of development is quite similar to that of the cAMP receptor 1 (*carA*), and appears to play an essential role in the initiation of differentiation and also spore maturation, closely related to the cAMP signaling system [15].

ρ^∆^ cells with a reduced amount (about 1/4) of mitochondrial DNA (mtDNA), derived from *D. discoideum* Ax-2 cells, exhibit greatly delayed differentiation and fail to construct fruiting bodies: prestalk differentiation is significantly enhanced inρ^∆^ slugs, while prespore differentiation is markedly inhibited [16]. Moreover, ρ^∆^ slugs also exhibit highly disordered phototaxis [17], thus indicating the necessity of a certain amount of mitochondrial genome DNA for normal development of *Dictyostelium* cells.

Importantly, a mitochondrial gene cluster (*dia3* consisting of *nad11*, *nad5*, *rps4*, *rps2*, and *nad4L*) including ribosomal protein S4 (*rps4*), are specifically expressed in response to starvation around the GDT point and play essential roles in the initiation of cell differentiation in Ax-2 cells. The *rps4* gene is present as a single copy in mt-DNA, but the copy number must be multiple, because numerous mitochondria are contained in a cell. In spite of this situation, we tried homologous recombination to determine the function of mt-RPS4, by inactivating the subpopulation of the *mt-rps4* gene [10]. As was expected, partial disruption of *mt-rps4* was found to cause impaired differentiation, thus resulting in the failure of many cells to aggregate even after a prolonged time of starvation [10]. Transformants (*rps4*^AS^ cells) generated by antisense-mediated gene inactivation also exhibit markedly delayed differentiation; most of them showed no sign of aggregation and remained as round-shaped single cells even after 16 h of incubation [18]. In contrast, the initial step of cell differentiation including aggregation under the submerged conditions was enhanced in *rps4*^OE^ cells overexpressing the *mt-rps4* mRNA in the extramitochondrial cytoplasm [10]. Here it is of interest to note that the antisense-*rps4* RNA synthesized in the extramitochondrial cytoplasm is effective as the partial disruption of *mt-rps4* gene. This seems to indicate that a trace of the mt-*rps4* mRNA and/or RPS4 protein, both of which are synthesized in mitochondria, may be released to the extramitochondrial cytoplasm. Alternatively, it is also possible that the antisense-*mt-rps4* RNA may enter mitochondria to inactivate *mt-rps4* mRNA. Based on PSORTII prediction, when the mt-RPS4 protein is released into the cytosol, it is predicted to move preferentially to the nucleus. It was confirmed by the immunohistochemical method using the anti-mt-RPS4 antibody that the mt-RPS4 protein produced in the cytoplasm of the *rps4*^OE^ cells is capable of moving into the nucleus [18]. Again, it is worth noting that the partial disruption (mitochondrial heteroplasmy) of the *rps4* gene greatly impairs differentiation, including cell aggregation. Although the fact that only the RPS4 protein of *Dictyostelium* has several nuclear localization signals is puzzling, at least a part of the mt-RPS4 protein seems to work in the nucleus to regulate cell differentiation. In other organisms, their mt-RPS4 proteins lack nuclear localization signals. It is generally difficult for proteins located in the mitochondrial matrix to go out to the cytosol, because mitochondria are partitioned by two (outer and inner) membranes. However, several mitochondrial proteins like apoptosis-inducing factor (AIF; [19]), endonuclease G (EndoG; [20]), and heat shock protein 70 (Hsp70; [21]) have been shown to move to the nucleus in response to apoptosis and heat shock. All of these proteins are encoded by the nuclear genome DNA, followed by translocation to the mitochondrion and then again to the nucleus. Accordingly, the behavior of *Dictyostelium* mt-RPS4 is unique in that it is encoded by the mtDNA.

**Figure 3:**
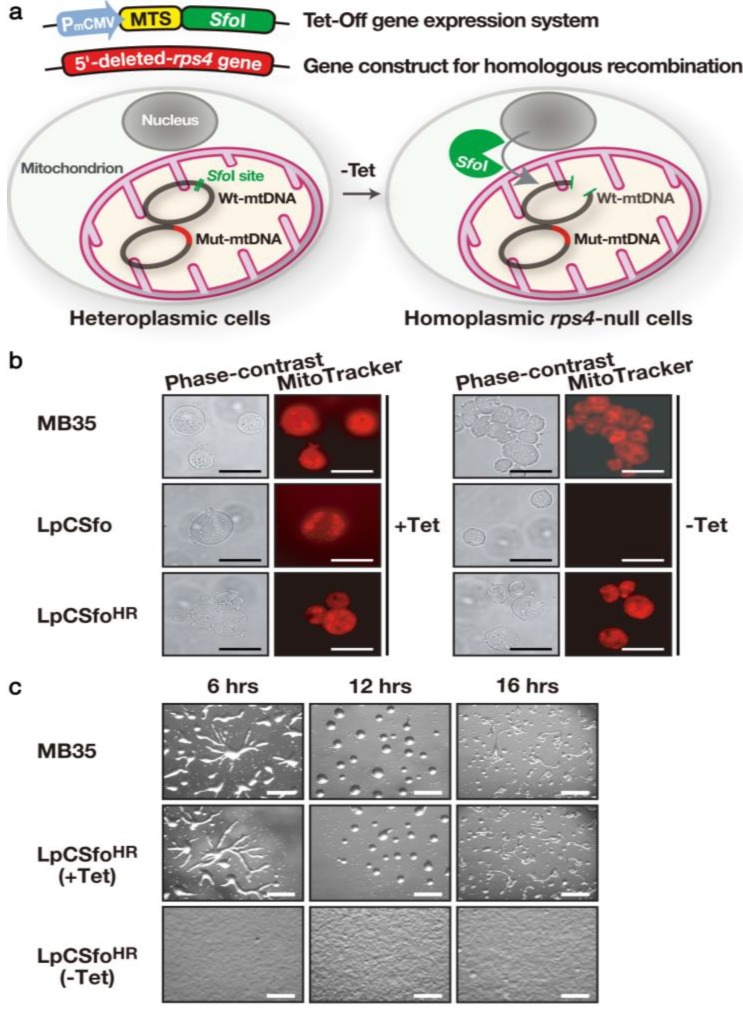
Strategy for creating *rps4*-null cells and their phenotypes. (**a**) As a starting material, LpCSfo cells in which pCoxIV (MTS)-*Sfo*I is expressed under the tetracycline-minus (−Tet) condition, were prepared. Subsequently, the mutant *rps4* gene (Mut-mtDNA for homologous recombination), in which the upstream *Sfo*I-site and a 5'-half of *rps4* coding region were deleted, was introduced into LpCSfo cells to obtain heteroplasmic transformants (LpCSfo^HR^ cells) with mitochondria consisting of the Mut-mtDNA and wild-type mtDNA (Wt-mtDNA). Coupled with removal of Tet from growth medium, the fusion protein MTS-*Sfo*I synthesized in the cytoplasm is exclusively transferred into mitochondria of LpCSfo^HR^ cells and selectively digests Wt-mtDNA but not Mut-mtDNA. Since the digested Wt-mtDNA is not duplicated, the Mut-mtDNA becomes dominant during the course of growth under the −Tet condition, thus eventually giving *rps4*-null cells. (**b**) These cells were grown in growth medium with (+Tet) or without (−Tet) teteracyclin. Membrane potential of mitochondria was visualized by staining of cells with MitoTracker Orange. As was expected, the staining of mitochondria was almost completely vanished in LpCSfo cells grown without Tet, because their mtDNA with an intact *Sfo*I site would be cleaved by *Sfo*I eventually to become a ρ^0^ state devoid of mitochondrial DNA. Bars, 200 nm. (**c**) Development of starved MB35 cells and LpCSfo^HR^ cells on agar. MB35 cells and LpCSfo^HR^ cells grown with (+Tet) or without (−Tet) tetracycline were washed twice in BSS and plated on 1.5% non-nutrient agar at a density of 5 × 10^6^ cells/cm^2^. This was followed by incubation for the indicated time at 22 °C. Bars, 0.5 mm. (Basically from Chida *et al*. [22]).

Provided that it is possible to completely inactivate *rps4* expression, the *rps4*-null cells should never differentiate from the GDT point in response to starvation. To test this, we have explored a new method for specifically disrupt a mitochondrial gene (*rps4*), by a combination of homologous recombination and delivery of an appropriate restriction endonuclease (*Sfo*I) into mitochondria [22]. First, mitochondrially targeted *Sfo*I (*i.e*., mitochondrial targeting sequence (MTS)-*Sfo*I fusion protein) whose expression is under control of the tetracycline (Tet)-regulated gene expression system was introduced into cells heteroplasmic with respect to the *rps4* gene. The heteroplasmic cells were produced by homologous recombination by use of the construct in which the unique *Sfo*I site and 5'-half of the *rps4* coding region were deleted not to be digested by *Sfo*I, and therefore their mitochondria have both the wild-type mtDNA and the mutant mtDNA with the disrupted *rps4* gene (Figure 3a). In response to removal of Tet from growth medium, *Sfo*I is selectively delivered into mitochondria and digests only the wild-type mtDNA but not the mutant mt-DNA, thus giving *rps4*-null cells with only the mutated mtDNA, under the Tet-minus condition. A starting material, LpCSfo cells in which pCoxIV (MTS of cytochrome c oxidase subunit IV)-SfoI is expressed under the Tet-minus condition stop growing in growth medium within 48 h after their transfer to Tet-minus medium, because they are converted to a rho-zero state in which mtDNA is completely eliminated (Figure 3b). However, LpCSfo^HR^(−Tet) cells (*rps4*-null cells) have almost the same number of apparently intact mitochondria and are able to grow normally with nearly the same doubling-time, as in the case of parental MB35 cells, indicating that the intact *rps4* gene is not required for growth. As expected, starving *rps4*-null cells (LpCSfo^HR^(−Tet) cells) exhibit a great delay of differentiation: no sign of cell aggregation is noticed even after 16 h of incubation, though LpCSfo^HR^(+Tet) cells show normal development (Figure 3c). This strongly suggests that the mt-RPS4 protein and/or *mt-rps4* mRNA may be essential for the initiation of cell differentiation. The novel method presented here must provide a powerful tool for precisely determining the functions of individual mitochondrial genes as well as for genetic therapy of mitochondrial diseases.

## 3. Mitochondrial Molecular Chaperon, TRAP-1, Is Necessary for the Initiation of Differentiation as Well as for Growth of *Dictyostelium* Cells

**Figure 4:**
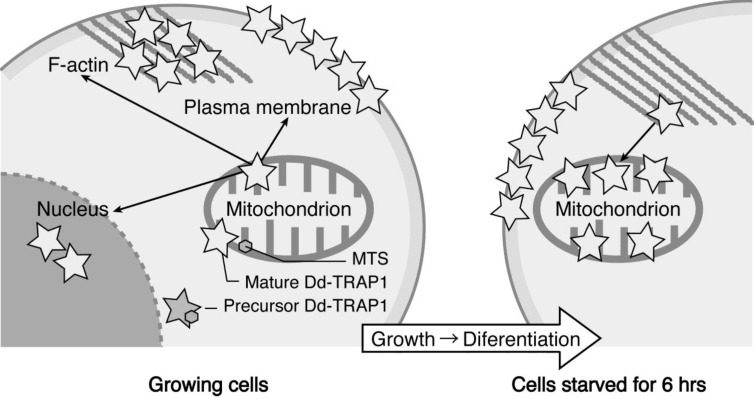
Schematic drawing showing the behavior of Dd-TRAP1 during the prestarvation response (PSR) and the initiation of differentiation in *Dictyostelium* cells (See the text for details).

We have demonstrated that the *Dictyostelium* homologue (Dd-TRAP1) of TRAP-1 (tumor necrosis receptor-associated protein 1), which is a molecular chaperone belonging to the heat-shock protein 90 (Hsp90) family, translocates from the cell cortex to mitochondria as the density of growing cells increases, which allows the prompt transition of cells from growth to differentiation through a novel prestarvation factor (PSF-3) in growth medium (Figure 4) [23,24]. When Ax-2 cells growing at low cell density (5 × 10^5^ cells/mL), in which Dd-TRAP1 is localized in the cell cortex, were harvested and incubated in conditioned growth medium in which Ax-2 cells had been grown up to the late exponential growth phase (and therefore contained a sufficient amount of PSF- 3), Dd-TRAP 1 was found to quickly translocate to mitochondria within 1 min of incubation even at low density. As described above, when cells growing at a high cell density (5 × 10^6^ cells/mL) are transferred to fresh growth medium, their differentiation competence, acquired by the prestarvation response (PSR), is lost within 30 min of incubation [25]. Similarly, when Ax-2 cells growing at the late exponential growth phase (8 × 10^6^ cells/mL) are transferred to fresh growth medium, Dd-TRAP1 located in mitochondria quickly returns to the cell cortex within 30 min of incubation in fresh growth medium [24].

Burdine and Clarke [26] have reported that prestarvation genes such as *discoidin I* and the 2.4-kb *PDE* (phosphodiesterase) are barely induced in *PKAcat* (cAMP-dependent protein kinase, catalytic subunit)-null cells, but their expression is normal in *Gβ*-null cells, suggesting that the PSR as assayed by *discoidin-I* expression is regulated by protein kinase A (PKA), but not by the G-protein β subunit. Importantly, the translocation of Dd-TRAP1 to mitochondria is observed both in *Gβ*-null cells and in *PKAcat*-null cells [24]. This indicates that neither PKA nor Gβ is required for the translocation via the novel PSF-3-mediated PSR. The Dd-TRAP1 seems to be essential for cell viability, because we failed to obtain *Dd-trap1*-null cells by means of homologous recombination. The knockdown mutant of Dd-TRAP1 (TRAP1-RNAi cells) exhibits a significant defect in PSR [24,27]. Although TRAP1-RNAi cells show normal expression of classic prestarvation genes such as *dscA* and *carA*, the expression of differentiation-associated genes (*dia1* and *dia3*) induced by the PSR is markedly repressed. In contrast, transformants overexpressing Dd-TRAP1 show an early PSR and also increased expression of *dia1* and *dia3* in a cell-density dependent manner in growth medium [24].

**Figure 5:**
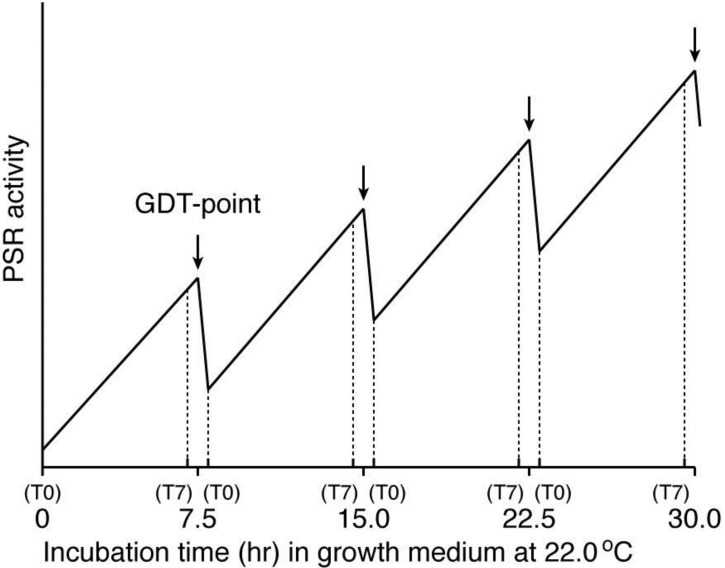
A working hypothesis for explaining the existence of the GDT-point in the *Dictyostelium* cell-cycle, by assuming a temporally oscillating PSR activity during synchronized cell growth. This model is based on the fact that there is a good correlation between the expression patterns of the prestarvation (PS) genes and the GDT point-specific genes, and is also constructed from the presumption that the amount of PSFs (*i.e*., PSF activity) in an ideally synchronized cell population may oscillate in a cell-cycle dependent manner during growth, as shown here. The cell-cycle positions of T0 and T7 offer themselves repeatedly during the course of a completely synchronized growth (see Figure 2). The PSR activity reaches the maximum value at the GDT-point (arrow between T7 and T0) in each cell cycle. Although it is presently unknown whether the expressions of Dd-TRAP1 and/or the amount of PSF-3 actually oscillate in a cell-cycle dependent manner, increasing their basal levels during the progression of synchronous cell growth, this issue remains to be examined in future. The horizontal axis of this figure represents incubation time (h) of an ideally synchronized *D. discoideum* (Ax-2) cell population in growth medium at 22.0 °C. T0 and T7 indicates 0 and 7 h, respectively, after a temperature shift from 11.5 °C to 22.0 °C for cell synchrony, and these are repeatedly represented during a prolonged time of synchronized culture.

*Dictyostelium* cells have mechanisms to sense when hard times such as starvation are approaching. There is a density-sensing mechanism (PSR) that is working during growth and prepares growing cells to induce the initial step of differentiation including the acquisition of aggregation-competence. At least three different kinds of PSFs (PSF-1, PSF-2, and PSF-3) are synthesized during growth and accumulate in the microenvironment such as growth medium according to the density of the cells. Although the precise roles and action mechanisms of these PSFs remain to be elucidated, they will be defined when pure PSFs, the specific antibodies against them, and the genes encoding them are available in further studies. Here it is of importance to note a good correlation between the prestarvation genes (PS-genes) and the GDT point-specific genes [3], thus suggesting that the maximum level of PSR activity may be a promising entity of the GDT-point, as shown schematically in Figure 5. This hypothetical idea remains to be inspected using highly synchronized cell populations in future studies.

TRAP-1 (Hsp75) was initially identified as a novel protein that binds to tumor necrosis factor 1 (TNF-1) [28], and several studies suggest that TRAP-1 plays roles in cell-cycle progression, cellular differentiation and apoptosis. In the Hsp90 family, TRAP-1 has a mitochondrial localization sequence at its N-terminus [28,29] and is located predominantly in mitochondria in several cell lines [29,30]. However, TRAP-1 has also been identified as an interacting partner for several extramitochondrial proteins (the type 1 TNF receptor, TNFR-1; the retinoblastoma protein, Rb; and EXT1, EXT2) [28,31,32]. In mammalian cells, extramitochondrial localization of TRAP1 has been observed in secretary granules, nuclei, and at the cell surface [30]. Mitochondrial Hsp60 has been found at a discrete extramitochondrial sites, including the cell surface, cytoplasmic vesicles and granules, peroxisomes, and ER [31]. In *Dictyostelium* cells, newly synthesized Dd-TRAP1 (80 kDa) with a mitochondrial targeting sequence (MTS) is translocated to the cell cortex after a brief and temporal stay in mitochondria [23]. Coupled with an increase of cell density at the growth phase, Dd-TRAP1 is again and rapidly translocated from the cell cortex to mitochondria by PSF-3-mediated PSR. The molecular mechanism by which the processed Dd-TRAP1 (73 kDa) devoid of MTS is able to come back to mitochondria is presently puzzling. Importantly, Ledgerwood *et al*. [33] have reported that exogenously added TNF is found to be delivered to mitochondria. This raises the possibility that in cells activated by TNF, TRAP-1 might transfer TNF itself and other related proteins from the cell surface to mitochondria.

It has been reported that decreased expression of TRAP-1 enhances age-dependent loss of *Drosophila melanogaster* dopamine (DA) and DA neuron number resulting from human [A53T]α-Synuclein expression, and also that it results in an enhanced loss of climbing ability of the fly and sensitivity to oxidative stress [34]. Meanwhile, overexpression of human TRAP-1 was able to rescue these phenotypes in *Drosophila*. Human TRAP-1 overexpression in rat primary cortical neurons also rescues [A53T]α-Synuclein-induced sensitivity to rotenone treatment, thus indicating that [A53T]α-Synuclein toxicity is intimately connected to mitochondrial dysfunction and that toxicity reduction in *Drosophila*, rat primary neurons and human cell lines can be achieved using overexpression of the mitochondrial chaperone TRAP-1 [34]. In TRAP-1 negative cells (A549: adenocarcinomic human alveolar basal epithelial cells) silenced by siRNA, there are high levels of cell proliferation promoting genes coding for G-protein coupled receptors, cell adhesion genes and genes associated with Rho-kinase pathways [35]. Levels of genes involved in cell motility and metastatic spread is also raised in the TRAP-1 negative cells, thus suggesting that in many tumors TRAP-1 is able to activate cell proliferation whilst inhibiting metastatic spread [35].

An increase of mitochondrial membrane permeability is one of the key events in apoptosis, because it leads to the release of mitochondrial apoptogenic factors, such as cytochrome c, into the cytoplasm that activate downstream target of apoptotic cell death. Bcl-2 family is one of the best-characterized proteins that directly regulate mitochondrial functions: A major role of the Bcl-2 family of proteins is to alter mitochondrial membrane permeability, thus controlling the release of caspase-activating cytochrome c. Recent reports have disclosed the involvement of TRAP-1, as one of apoptogenic regulators other than Bcl-2. Downregulation of TRAP-1 expression enhances the release of cytochrome c from mitochondria. Since reactive oxygen species (ROS) are closely involved in the regulation of the TRAP-1 expression, it is most likely that TRAP-1 is a critical sensor involved in ROS-mediated regulation of apoptosis [36]. TRAP-1 has been recently demonstrated as a component of a mitochondrial pathway selectively up-regulated in tumor cells which antagonizes the proapoptotic activity of cyclophilin D and is implicated for the maintenance of mitochondria integrity, thus favoring cell survival. Importantly, novel TRAP-1 antagonists are shown to cause sudden collapse of mitochondrial functions and selective tumor cell death, suggesting that this pathway may represent a novel molecular target to improve anticancer therapy. Moreover, TRAP-1 is significantly up-regulated in human osteosarcoma cells adapted to growth in mild oxidizing conditions, and this correlates with a phenotype more resistant to H_2_O_2_-induced DNA damage and apoptosis [37]. This seems to indicate a possible role of TRAP-1 as being responsible for multi-drug resistance in human colorectal cancer: TRAP-1 may give us a potential therapeutic target which has important implications for the efficacy of anticancer agents in cancer therapeutics.

## 4. Cyanide-Resistant Respiration and Cell Differentiation

We have previously reported that benzohydroxamic acid (BHAM), a potent inhibitor of cyanide (CN)-resistant respiration mediated by alternative oxidase (AOX), induces formation of unique cell masses (*i.e.*, stalk-like cells with a large vacuole and thick cell wall) in starved *Dictyostelium* cells [38]. Another inhibitor of CN-resistant, propyl gallate (PG) also has essentially the same effect with BHAM [38]. Unexpectedly, however, *aox*-null cells prepared by homologous recombination exhibited normal development under normal culture conditions on agar, indicating that BHAM-induced stalk formation is not solely attributable to inhibition of CN-resistant respiration [39]. This suggests that a series of pharmacological approaches in the field of life science has serious limitations. Thus, it is most likely that BHAM induces stalk formation through unknown side effect(s) on *Dictyostelium* cells, coupled with its ability to block the AOX pathway. It has been shown that differentiation-inducing factors (DIFs) are closely involved in stalk cell differentiation [40], and that DIF-1 is the inducer of PstO cells, a subtype of prestalk cells [41]. Importantly, a rise in cytoplasmic free Ca^2+^ and H^+^ concentrations triggers stalk cell differentiation, possibly by mimicking the roles of DIF-1 [42]. Accordingly, it is of interest to know if BHAM affects the intracellular DIFs, Ca^2+^- and/or H^+^-concentrations.

Under stress (e.g., in submerged culture), starved *aox*-null cells exhibited slightly delayed aggregation compared with parental Ax-2 cells; most cells remained as loose aggregates even after prolonged incubation [39]. CN-resistant respiration may cushion cellular damages through ROS, because the developmental defects of *aox*-null cells became more marked when they were incubated for 30 min just after starvation in the presence of more than 1.75 mM H_2_O_2_: most cells were round and floating on water. This seems to indicate that CN-resistant respiration could mitigate cellular damage through reactive oxygen species (ROS), because AOX has a potential role in reduction of ROS production. Starved *aox*-null cells did not develop in the presence of 5 mM KCN (which completely inhibited the conventional cytochrome-mediated respiration) and remained as non-aggregated single cells on agar even after prolonged incubation. Somewhat surprisingly, however, parental Ax-2 cells were found to develop normally, forming fruiting bodies even in the presence of 10 mM KCN. Taken together, these results suggest that CN-resistant respiration might compensate for the production of ATP via oxidative phosphorylation.

**Figure 6:**
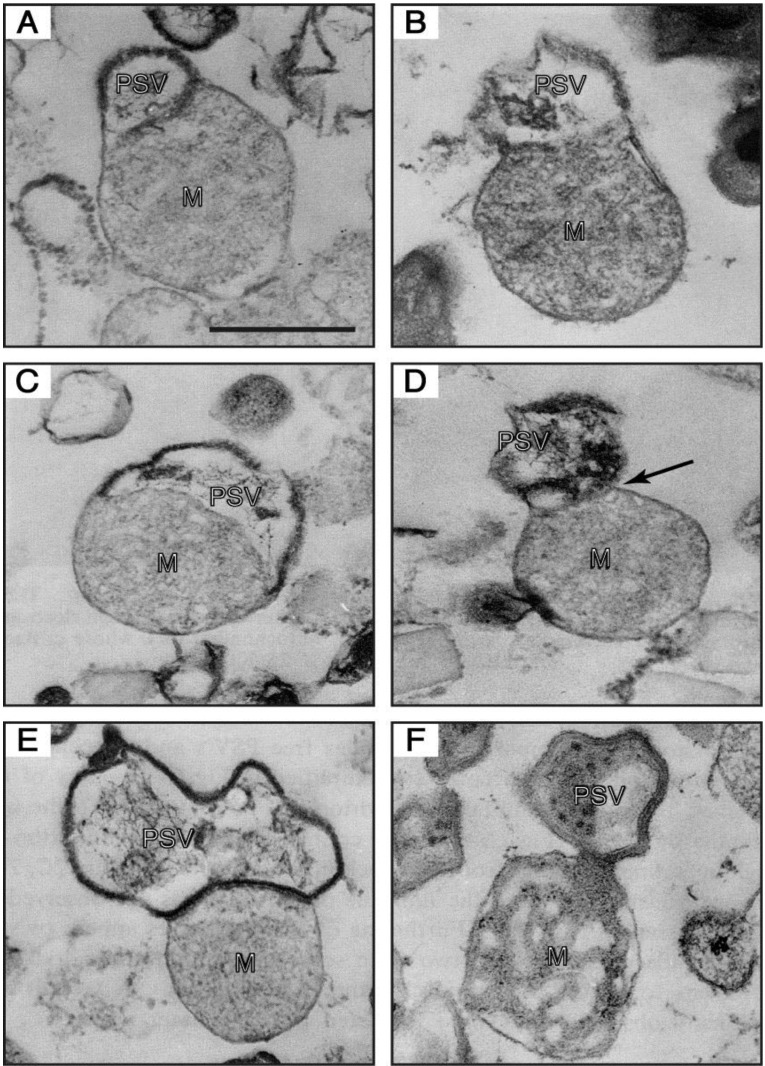
Electron-micrographs of PSV-mitochondrion complexes present in a 47.5%–50% fraction (the intermediate fraction between a lighter pure mitochondria and a heavier pure PSV fractions) of a multilayered sucrose gradient for cell fractionation. (**A**,**B**) the outer membrane of a mitochondrion (M) is continuous with the unit membrane of a PSV. (**C**) a PSV and a mitochondrion are partitioned by a single membrane derived from either the PSV or the mitochondrion, and the electron-dense lining membrane of the PSV is not observed at the contact region. (**D**) a PSV appears twisted at the contact region (arrow). (**E**) a completely formed PSV is in close contact with a mitochondrion. (**F**) a mitochondrial part itself loses its inner structural integrity and seems to be transformed into the PSV. Bar, 500 nm. (From Maeda [49]).

Although the expression of *Dictyostelium* AOX protein is downregulated at the stationary phase in growth medium, the level of AOX protein increases upon starvation and remains at least 10 times higher compared with cells at the stationary growth phase [43,44]. Accordingly, AOX has been proposed to play a critical role in starvation-induced events including cell differentiation, relating to the ability of energy-dissipating systems to reduce the production of ROS (*i.e*., the initiators of apoptosis *in vivo* and *in vitro*) [45].

The presence of cyanide in culture medium is shown to markedly retard the growth of the alo1 ⁄ alo1 mutant of *Candida albicans*, which is devoid of D-arabinono-1,4-lactose oxidase (ALO) that catalyzes the final step of D-erythroascorbic acid (EASC) biosynthesis. The increased EASC level is shown to induce the expression of AOX [46]. Also, the respiratory inhibitors that block the conventional cytochrome pathway are known to induce the expression of AOX [47]. AOX could compensate for the production of ATP via oxidative phosphorylation. In general, however, AOX is believed to be efficient in free energy dissipation by decreasing the yield of oxidative phosphorylation during state 3 respiration: AOX can consume the reducing power by substrates without energy conversion in to an H^+^ electrochemical gradient (energy source of ATP synthesis). Therefore, the precise mechanism of the role of AOX in cell survival and development, particularly under stressed conditions, is presently unknown and remains to be elucidated.

## 5. A Cell-Type Specific Structure (PSV: Prespore-Specific Vacuole) Is Formed by Mitochondrial Transformation in Cooperation with the Golgi Complex

**Figure 7:**
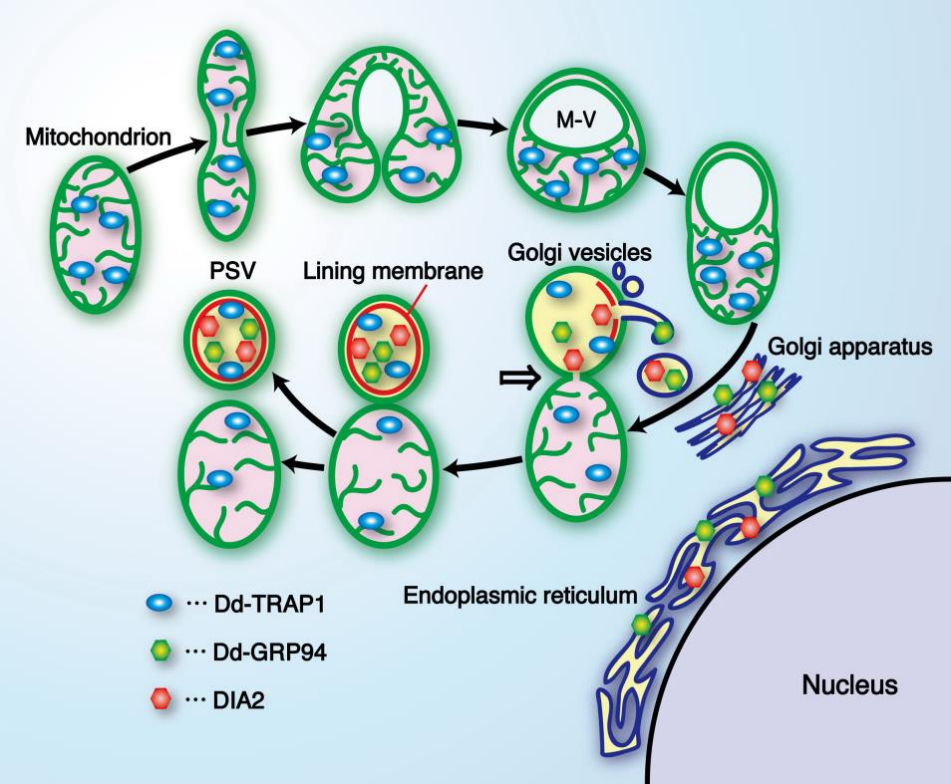
A diagrammatic representation showing formation of the prespore-specific vacuole (PSV) from a mitochondrion and Golgi vesicles. Prior to PSV formation, mitochondria in differentiating prespore cells undergo drastic transformation to form a sort of vacuole (M vacuole), and some Dd-TRAP1 molecules translocate into the M vacuole. Subsequently, Golgi vesicles containing DIA2, Dd-GRP94 and other materials required for PSV formation fuse with the M vacuole, thus resulting in formation of the lining membrane (red) and the internal fibrous structure in the M vacuole. The mitochondrion-PSV complex is eventually twisted at the junction (arrow) and detached to form the respective organelles. Interestingly, almost all of the DIA2 molecules are selectively translocated to PSVs (possibly M vacuoles) in differentiating prespore cells, and seem to be required for exocytotic secretion of PSVs to form the outer-most membrane of spore cell wall [15]. M-V, M vacuole; GRP94, glucose-regulated protein 94 (endoplasmic reticulum Hsp90). (Slightly modified from Maeda [12]).

The PSV (prespore-specific vacuole) is the sole organelle that is formed only in differentiating prespore cells but never in prestalk cells [48], and therefore elucidation of the precise mechanism of the PSV genesis is of particular importance for understanding the structural basis of cell or organelle differentiation. Quite interestingly, the mitochondrion undergoes a drastic transformation to form a unique vacuole (M-vacuole) just before in prespore differentiation, and this was followed by a mature PSV in it. Such drastically transforming mitochondria and PSV-mitochondrion complexes were actually observed in differentiating prespore cells, and also a considerable number of PSV-mitochondrion complexes as shown in Figure 6 were found in the intermediate fraction between a pure PSV and a pure mitochondria fractions, which were obtained by isopicnic centrifugation of cellular components of the prespore cell through a multilayered sucrose density gradient [49]. Based on our previous studies [49,50,51,52,53,54], it is evident that PSVs are constructed from mitochondria-derived M-vacuoles with the help of the Golgi complex, as schematically shown in Figure 7. That is, mitochondria in differentiating prespore cells are remarkably expanded, bent and fused at the ends to form a sort of vacuoles (M vacuoles) prior to PSV formation, as described above, and several Dd-TRAP1 molecules translocate into the M vacuoles. Subsequently, Golgi vesicles containing DIA2, Dd-GRP94 (*Dictyostelium* homologue of glucose-regulated endoplasmic reticulum Hsp90; [54,55]) and other molecules required for PSV formation fuse with the M vacuole, thus resulting in formation of the lining membrane and the internal fibrous structure in the M vacuole. The mitochondrion-PSV complex is eventually twisted at the junction and detached to form the respective organelles (Figure 7). It has been cytochemically determined that the activities of succinic dehydrogenase and cytochrome c oxidase, typical mitochondrial enzymes, are specifically detected in the lining membrane of PSV as well as in mitochondria [49]. Also, it has been demonstrated immunoelectoron microscopically that a monoclonal antibody specific to the lining membrane of PSVs stains a limited part of the mitochondria adjacent to the PSV- mitochondrion complexes [43]. On the other hand, a Golgi origin of PSVs has been proposed by both immunoelectron microscopy with polyclonal anti-spore IgG and electron microscopic autoradiography with [3H]-fucose, which is specifically incorporated into prespore cells [52]. Furthermore, an electron-dense membraneous structure similar to the lining membrane of PSV is sometimes observed in the Golgi cistaenae in prespore cells differentiating in a liquid shake culture [51]. Thus it is clear that both mitochondria and Golgi complexes are cooperatively implicated for PSV formation. The PSV is a functionally essential structure and it is exocytosed from prespore cells to form the outermost layer of spore cell wall during culmination [56,57], thus giving spores the physico-chemical strength.

Here, it is of interest to note that a reduced amount of mtDNA impairs greatly and specifically prespore differentiation [16], and that a *Dictyostelium* homologue (Dd-TRAP1) of TRAP-1, mitochondrial Hsp90, is closely involved in spore maturation as well as in the prestarvation response (PSR) [23,24,54].

Yolk crystal inclusions that distribute unevenly along the axis of animal-vegetal pole are already known to be formed in transformed mitochondria during oogenesis in a wide range of animal species [58,59]. The mitochondria of plant cells sequentially develop crystal dilatations, spherical intracrystal inclusions, and eventually crystalloid inclusions within their cristae, all apparently in response to a series of cytoplasmic stimuli [60]. A drastic structural change of mitochondria has been also reported in the plant egg cells of *Pelargonium zonale* [61]. The marked similarities of these specializations in the usual mitochondrial form within highly diverse situations probably reflect the universality of these modes of mitochondrial structural and functional plasticity.

## 6. Involvements of the Mitochondrial Functions in a Variety of Developmental Processes

Mitochondria exhibit different abundances, morphologies and functions in different cell types that adapt in response to environmental and cellular states. The cytopathology of mitochondrial diseases is usually caused by severe cellular ATP depletion due to mtDNA deletion. In *Dictyostelium*, mitochondrial large ribosomal RNA (mtlrRNA) is required for normal phototaxis and thermotaxis of a migrating slug [17]. It has been shown that the mitochondrial function is also impaired by mutations affecting nuclear-encoded proteins required for correct folding in the organelle of both mitochondrially- and nuclear-encoded proteins, and that antisense RNA inhibition of the expression of chaperonin 60, one of such proteins, impairs signal transduction for phototaxis of *Dictyostelium* slugs [62]. ATP-activated protein kinase (AMPK) is a ubiquitous, highly conserved protein that maintains cellular energy homeostasis of cells, as a highly sensitive energy sensor that is stimulated by ATP-depleted stresses [63]. Although phagocytosis and macropinocytosis consume chemical energy like ATP, they are not affected by mitochondrial disease and AMPK expression levels [63]. Interestingly, however, aberrant phenotypes in mitochondrially diseased cells, such as impaired phototaxis and thermotaxis, are completely suppressed by antisense-inhibiting AMPK expression, thus suggesting that diverse cytopathologies in *Dictyostelium* mitochondrial disease may be caused by chronic AMPK signaling not by insufficient ATP [64]. Recently, Carilla-Latorre *et al*. [65] have demonstrated that MidA, one of homologue proteins of unknown function named DUF185, is putative methyltransferase that is required for mitochondrial complex I (CI) function: a *Dictyosteliim* *midA*^−^ mutant exhibits a complex phenotypic outcome, which includes phototaxis and thermotaxis defects, and these phenotypes are mediated by a chronic activation of AMPK, indicating a possible role of AMPK signaling in CI cytopathology. With respect to chemotaxis, a novel mitochondrial protein (Tortoise) has been shown to be essential for directional movement of *Dictyostelium* cells in cAMP gradients [66].

In *Drosophila*, the embryonic body axes are specified during oogenesis, when cytoplasmic determinants localize to different regions of the developing oocyte. This gives the formation of positional information centers, which define polarity and pattern of the body plan along the anterior-posterior (AP) and dorsal-ventral (DV) axes during embryogenesis. The germ cell line is determined during embryogenesis by the large subunit of mitochondrial ribosomal RNA (mtlrRNA) [67,68]. The molecular function of the two factors in *Drosophila* embryos, mt-rRNAs and Nanos protein, which are required for the formation of and differentiation of the germline progenitors, respectively [69]. Mitochondrial ribosome protein L34 (mRpL34) is involved in ribosomal protein translation, encoded by a class of mitochondrial genes responsible for mitochondrial diseases that typically lead to muscle and brain disorders, and regulates differentiation in the *Drosophila* eye, coupling with the expression of dystroglycan (DG) [70]. A point mutation in the *Drosophila* gene *technical knockou*t (*tko*), encoding mitochondrial protein S12, causes a phenotype of respiratory deficiency, developmental delay, and neurological abnormalities similar to those presented in many human mitochondrial disorders, as well as defective oocyte behavior [71,72], and also the detailed patterns of gene expressions in various situations were reported, using a *Drosophila* model of mitochondrial disease [73]. Recently, mitochondrial fission factor (DRP1; dynamin-like protein) has been shown to be essential for follicle cell differentiation during *Drosophila* oogenesis [74] as well as for embryonic development and synapses formation in mice [75]. In this connection, it is of interest to note that mitochondrial DNA replication and/or cell division in *Dictyostelium* seems to be required for prespore differentiation, *i.e*., PSV formation [76,77,78].

The mitochondrial respiratory complexes have been generally considered as major producers of cellular ROS that may assist to fight viral or bacterial invaders or act as a signaling molecule. Under pathological conditions, however, the activation of NADPH-derived ROS is implicated in a number of metabolic diseases. Recently, the phagocyte-like NADPH oxidase (PHOX) was found to promote cytokine-induced mitochondrial dysfunction in mammalian pancreatic β-cells, leading to the release of cytochrome c activation of caspase 3, and cell death: the mitochondrial membrane potential is markedly lost by PHOX [79]. This finding will provide a promising field to link “mitochondrial defects” with functional studies on mitochondria, glucose-stimulated insulin secretion, and apoptosis.

Immature mitochondria of early embryonic organs (e.g., heats) must build more complex structures to ensure proficient and energetically competent ones. A recent report insists mitochondria, and more specifically the mitochondrial permeability transition pore (mPTP), as gating mechanism underlining cell differentiation in the developing heart, implicating cross-talk between genetic and metabolic signaling: the mPTP actually controls cardiac mitochondrial maturation and myocyte differentiation [80]. In this connection, knockout of cytophilin D, a member of the mPTP component, has been shown to result in elevated mitochondria matrix Ca^2+^, which enhances the activation of Ca^2+^-dependent dehydrogenase reducing metabolic flexibility [81]. Mammalian 16S mitochondrial rRNA is localized in the nucleus during spermatogenesis, suggesting an association of the mitochondrial transcript with the meiotic chromosome in mouse and human testis [82]. In this case, the possibility that the 16S mitochondrial rRNA found in the nucleus is the result of nuclear transcription of a mitochondrial pseudogene was ruled out. Therefore, the 16S mitochondrial rRNA seems to be transferred from the organelle to the nucleus of speamatogenic cells by means of an unknown mechanism of RNA translocation. This also suggests that the mitochondrial membrane may be somewhat leaky to release some intramitochondrial molecules such as rRNAs, and more importantly that some of *Dictyostelium* mt-*rps4* mRNA and/or RPS4 protein (mt-RPS4) may be able to go out from mitochondria and ready to be transferred into the nucleus, because *Dictyostelium* mt-RPS4 has several nuclear-localization sequences in the molecule, as noted before [10,18].

In general, pluripotent cells contain fewer and immature mitochondria with poorly developed cristae and preferentially use anaerobic metabolism (glycolysis) for their energy supply, but differentiated cells tend to rely mainly on oxidative phosphorylations to produce ATP. Stem cells are defined by two key characters: self-renewal (*i.e*., the ability to proliferate without lineage commitment) and the capacity to differentiate into one or more specialized cell types. Based on their pluripotency degree, stem cells are categorized into at least four types: embryonic stem cells (ESCs), somatic stem cells (SSCs), induced pluripotent stem cells (iPSCs), and cancer stem cells (CSCs). The mitochondria of differentiated cells display a larger morphology and more distinct cristae, and these changes accompanied by an increase in the ATP content and ROS levels [83]. Antimycin A, a molecule blocking the electron flow in the complex III of the mitochondrial electron transport chain, is known in ESC differentiation to inhibit heart cell differentiation, while the use of complex II and IV inhibitors (thenoyltrifluoroacetate and KCN) does not prevent cardiomyocyte differentiation [84]. These results suggest that the activity of complex III is essential, while those of complex II and IV are dispensable, for heart cell differentiation. Also, spontaneous intracellular Ca^2+^-oscillations in muscle ESC cells is inhibited by antimycin A treatment, and a pulse of ionomicin (an ionophore used to raise the intracellular Ca^2+^ level), given at an appropriate time, are able to restore cardiomyocite differentiation. Accordingly, it is most likely that mitochondrial complex III activity may be required for the differentiation of cardiomyocites through its involvement in Ca^2+^-oscillations [84]. Supporting the notion that stem cell mitochondria are involved in their stemness status, the reprogramming of adult cardiomyocites toward a progenitor-like state is shown to occur during their partial fusion with human ESC cells (hMSCs), depending on the transfer of stem cell mitochondria into cardiomyocites [85]. Regarding ROS, both increased and decreased ROS levels, depending on the differentiation type studied, seem to be implicated for MSC differentiation. Interestingly, the mitochondrial phenotype observed in pluripotent cells may not only a feature of normal stem cells, but may also be used as a promising indicator of cancer stem cells (CSCs). Actually, lung CSCs (LCSCs) isolated from the A549 lung cancer cell line are characterized by mitochondria with a perinuclear arrangement and a low amount of mtDNA, consume less oxygen and have reduced levels of ATP and ROS compared with non-LCSCs [86], suggesting that mitochondrial and energy-metabolic features might be used to determine the “stemness” of both normal and cancer stem cells. Quite importantly, however, highly selective inactivation or elimination of CSCs must be essential to rescue human beings from tumor progression, because CSCs seem to be ultimate origins of individual tumor cells.

The proper mitochondrial function is essential for ESC proliferation, regulating differentiation, and suppressing the emergence of tumorigenic cells during the process of differentiation, thus suggesting the existence of an interplay mitochondrial biogenesis and stem cell defferentiation [87]. Under self renewing conditions, attenuating mitochondrial function makes ESCs more glycolytic-dependent, and it is associated with an increase in mRNA reserves of master “stemness” regulators such as Oct4, Nanog, Sox2, while attenuating mitochondrial function results in repression of these gene expressions [88]. Moreover, differentiation potential is compromised by abnormal transcription of multiple Hox genes [88]. This indicates that tumogeneric cells continue to persist under differentiation conditions in which mitochondrial function is attenuated. Interestingly, although ESCs and SSCs are normal stem cells, iPSCs (mature adult cells such as fibroblasts) have been shown to be artificially reprogrammed to an ESC-like state by overexpressing Oct4, Nanog, Sox2, KLF-4 and/or c-Myc, suggesting a close interplay between “stemness”, stem cell differentiation and mitochondrial biogenesis [89,90,91].

The PTEN-induced kinase 1 (PINK1) is a mitochondria-targeted serine/threonine kinase, which is linked to autosomal familial Perkinson’desease. Functional studies have demonstrated that PINK1 recruits Parkin to mitochondria to initiate the mitophagy, and that PINK1 is posttranslationally processed, whose level is definitely regulated in healthy steady state of mitochondria [92]. Thus PINK1 plays a crucial role in mitochondrial healthy homeostasis. Recent analysis of cardiac cells has also revealed that interaction between cytoskeletal proteins (such as beta-tubulin II) and mitochondria actively participate in mitochondrial regulation, and that potential candidates for the key role of this regulation are the cytoskeletal proteins plectin and tubulin [93]. In the heat, colocalization of beta-tubulin isotype II with mitochondria has been suggested to participate in the coupling of ATP-ADP translocase (ANT), mitochondrial creatine kinase (MtCK), and VDAC (ANT-MtCK-VDAV), contributing to maintenance of mitochondrial and cellular physiology [93].

**Table 1:** Involvements of various mitochondrial functions in *Dictyostelium* development.

Molecule, structure or metabolism	Function	Reference
**mitochondrial ribosomal protein subunit S4(mt-RPS4)**	The expression is essential for the initiation of cell differentiation from the DGT-point.	[9,10,18,22]
**mitochondrial DNA(mtDNA)**	Rho-delta (ρ∆) cells with a reduced amount (about 1/4) of mtDNA exhibit greatly delayed differentiation and fail to construct fruiting bodies.	[16]
**mitochondria-localized molecular chaperone(TRAP1)**	Its translocation into mitochondria induces the prestarvation response and subsequent cell differentiation.	[23,24,27,54]
**Tortoise (TorA)**	This novel mitochondrial protein is required for the efficient chemotaxis toward cAMP.	[66]
**mitochodrial large ribosomal RNA(mtlrRNA)**	The inactivation of mtlrRNA impaires photo-taxis and thermotaxis of the migrating pseudoplasmodium (slug).	[17]
**prespore-specific vacuole (PSV)**	This cell-type specific organelle is formed from a mitochondrion as the structural basis in alliance with the Golgi apparatus.	[3,12,53]
**cyanide (CN)-resistant respiration**	This AOX (alternative oxidase) activity is favorable for cell survival under stressed conditions.	[39]

## 7. Concluding Remarks

Several important roles of *Dictyostelium* mitochondria in cellular development are summarized in Table 1. Among these, the process of PSV formation is particularly amazing in that one of the most remarkable structures (organelles) which characterizes most strikingly the cell type is constructed by remarkable structural transformation of mitochondria and its cooperation with the Golgi apparatus. This process seems to be remarkably complex, but is quite interesting because it illustrates a highly cooperative interaction between certain organelles such as mitochondria and Golgi vesicles. The developmental roles of *Dictyostelium* mt-RPS4 and Dd-TRAP1 are also notable. Although most of the molecular mechanisms by which cell differentiation is controlled in a mitochondria-dependent manner remain to be elucidated, the new world of mitochondria with many essential functions beyond our imagination is slowly but surely spreading every day. We hope that the data presented in this review will offer insightful suggestions on the weight of mitochondrial functions in cell-cycle progression, differentiation and pattern formation as well as in the field of cancer research.
